# Cavity-Mediated Collective Resonant Suppression of Local Molecular Vibrations

**DOI:** 10.1021/acs.jpclett.5c01124

**Published:** 2025-06-12

**Authors:** Vasil Rokaj, Ilia Tutunnikov, H. R. Sadeghpour

**Affiliations:** † Department of Physics, 8210 Villanova University, Villanova, Pennsylvania 19085, United States; ‡ ITAMP, Center for Astrophysics, Harvard & Smithsonian, Cambridge, Massachusetts 02138, United States

## Abstract

Recent advances in polaritonic chemistry suggest that chemical reactions can be controlled via collective vibrational strong coupling (VSC) in a cavity. In this fully analytical work, we demonstrate that the collective vibrations of a molecular ensemble under VSC execute a beating with a period inversely proportional to the collective vacuum Rabi splitting. Significantly, this collective beating is imprinted on the local dynamics and resonantly suppresses individual molecular vibrations when a fraction of molecules is vibrationally excited, as in activated complexes formed in chemical reactions. This emergent beating occurs on significantly longer time scales than the individual molecular vibration or the cavity field oscillation period, peaking at the cavity-molecule resonance, consistent with polaritonic chemistry experiments. The cavity mediates an energy exchange between excited and ground-state molecules, affecting the dynamics of the entire ensemble. These findings suggest that the dynamics in polaritonic chemical reactions may not be in full equilibrium. In the ultrastrong coupling regime, we find that the local vibrations are modified by the cavity even at short time scales. Notably, these dynamical effects also extend to isotropic molecular ensembles in our model. Our analytical model offers insights into how collective VSC can dampen local molecular vibrations at resonance, potentially altering chemical reactivity.

## Graphical abstract

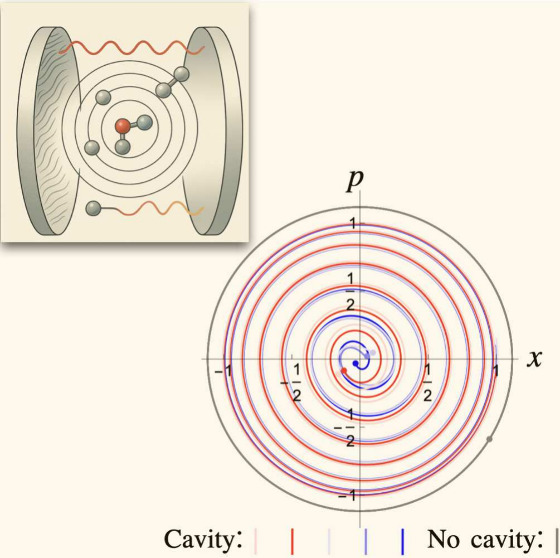

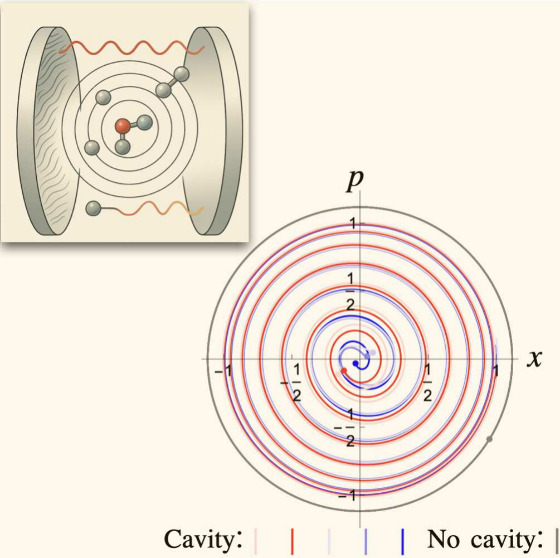

**Introduction.** Polaritonic chemistry has emerged as an appealing branch of synthetic chemistry that promises mode selectivity and a cleaner approach to controlling chemical kinetics.^1,2^ Experiments have suggested the control of chemical reactions through collective vibrational strong coupling (VSC) in ensembles of *N* molecules (*N* ≈ 10^6^ to 10^12^) in microcavities, without external fields,^3–8^ finding that optimal modification of reactivity occurs when the cavity is in resonance with molecular vibrational modes.^3–8^

These experimental findings have led to an extensive body of theoretical and experimental research, with the aim of gaining a mechanistic understanding of resonant VSC for chemical reactivity.^9–17^ Despite such efforts, the fundamental mechanisms behind the observed phenomena are poorly understood,^1^ primarily because of the inherent complexity and out-of-equilibrium nature of reaction dynamics.

One key challenge is to elucidate how a single-mode cavity can modify the local molecular dynamics in a large molecular ensemble under collective resonant VSC.^1^ From an equilibrium perspective, for example in transition state theory,^18^ this seems counterintuitive since in polaritonic systems, as, e.g., described by the Tavis-Cummings^19^ or the Hopfield model,^20,21^ there are two polaritonic modes and *N* – 1 dark states whose energies are unaffected by the cavity.^9,10^ The thermodynamic behavior at room temperature should therefore be dominated by the dark states and not affected by the cavity.^9,10,22^

In this work, we provide a fully analytical description of the out-of-equilibrium dynamics of a molecular ensemble under VSC, by evolving fully or partially excited ensemble configurations under collective coupling to a cavity. Our analytical solutions reveal the emergence of a resonant collective beating,^21^ which imprints itself on the local vibrational dynamics and resonantly suppresses individual molecular vibrations. The beating observed in our model arises from the interference between two polariton frequencies, which emerge when molecular vibrations are resonantly coupled to the cavity mode. This interference produces a fast and a slow oscillating mode, with the slowly oscillating mode defining the beating frequency.

The period of the emergent beating that suppresses the local molecular vibrations in an activated complex, is inversely proportional to the collective vacuum Rabi splitting (VRS), and thus depends on the number of coupled molecules. These findings imply that (i) the VRS introduces a global dynamical time scale that depends on the collective properties of the coupled system, rather than merely on the time scales of the subsystems; and (ii) the dynamics of molecular ensembles under VSC may not necessarily operate in equilibrium.

The beating period exhibits a pronounced peak around the cavity-molecule resonance, similar to the resonance dependence found in polaritonic chemistry experiments.^3–8^ This phenomenon underlies the resonant suppression of local vibrations in our model. It is worth noting that we describe the cavity-induced resonant beating in a classical framework, providing a clear intuition that resonant dynamical modifications can be observed at the classical level. The quantum nature of the observed phenomena in polaritonic chemistry remains an active research endeavor.^15,23–27^ Recent works suggested that the sharp resonance effect in polaritonic chemistry is purely quantum.^23–27^ Our findings within a classical formalism help shed light on the mechanisms for resonant suppression and opens a path for understanding resonant collective phenomena in polaritonic chemistry with classical dynamics simulations. It is important to note that the classical description of vibrational dynamics in our model exactly reproduces the quantum dynamics of the first moments due to Ehrenfests theorem for harmonic systems.^28,29^

In the ultrastrong coupling regime, where the counter-rotating terms in the Hamiltonian are significant,^30–32^ we find that the local molecular vibrations are modified by the cavity even on a short time scale. In this case, the molecular vibrations are strongly affected even during a single oscillation period, and the molecular dynamics become more complicated and do not follow a simple beating pattern.

Crucially, the cavity-induced modifications of the local vibrations require only a partially activated molecular ensemble (where ∼1–5% of the molecules are excited). This holds both in the strong and in the ultrastrong coupling regimes. Additionally, in the partially excited ensemble, the initially excited molecules transfer their energy to the ground-state ones. Thus, the cavity mediates an energy exchange between the excited and the ground-state molecules. These important dynamical phenomena in our model can also be generalized to the case of isotropic molecular ensembles. The fact that full activation or perfectly oriented molecules are not necessary, makes the proposed mechanism relevant for realistic activated complexes, thus potentially enabling alteration and control of chemical reactivity.

**Model Hamiltonian.** We consider a system of *N* identical noninteracting polar molecules collectively coupled to a single-mode cavity. The molecular vibrations are modeled using a one-dimensional harmonic potential. For simplicity, the molecules are assumed to be perfectly oriented along one of the cavity polarization directions. As we will show later, our model and analysis naturally extend to isotropic ensemble where half of the molecules are oriented with the field polarization and half are oriented against it. This system is described by the Pauli-Fierz Hamiltonian in the length gauge (ℏ = 1),^9,33,34^

1 $$Ĥ={Ĥ}_{0}-\frac{\omega \partial_{q}^{2}}{2}+\frac{\omega }{2}{\left(\mathrm{q̂}-g\overset{N}{\underset{i=1}{\sum }}{\mathrm{x̂}}_{i}\right)}^{2}$$

In the above, the Hamiltonian ${Ĥ}_{0}=\overset{N}{\underset{i=1}{\sum }}\left[-\partial_{x_{i}}^{2}/\left(2m\right)+m\Omega_{v}^{2}{\mathrm{x̂}}_{i}^{2}/2\right]$ describes the uncoupled *N* molecules, with Ω_v_ being the equilibrium frequency of the harmonic molecular potential, and *m* is the molecular mass. The operators *x̂*_*i*_ and – i∂_*x*_*i*__ correspond to the coordinates (bond lengths) and momenta of the molecules. In addition, ω = *π c*/*L* is the fundamental frequency of the cavity, and *c* is the speed of light in vacuum. Here, we assume a standard Fabry-Pérot cavity with effective optical volume V=AL , where A is the effective cross-sectional area of the confinement volume, and *L* is the distance between the cavity mirrors.^10^ The dimensionless light-molecule coupling constant is $g=\mu_{0}/\sqrt{\omega \epsilon_{0}V}$ , depending on V , the vacuum permittivity ϵ_0_, and the magnitude of the molecular dipole moment μ_0_ in units of elementary charge *e*. The operators *q̂* and – i∂_*q*_ describe the position and momentum quadratures of the cavity mode.^9,34^

The model Hamiltonian Ĥ has been used in multiple publications considering molecular systems under VSC in cavities.^9,10,35–37^ The oriented molecules couple to the cavity through the collective dipole moment operator μ̂ = μ_0_∑_*i*_ *x̂*_*i*_. This suggests that only one collective degree of freedom is coupled to the cavity. To show this explicitly, we express Ĥ in terms of the collective coordinate *X̂* = *N* ^–1/2^ ∑_*i*_ *x̂*_*i*_ and the relative bond lengths x~^j=N−1/2(x̂1−x̂j) with *j* = 2, ..., *N*. The prefactor *N* ^–1/2^ is introduced for mathematical convenience as in refs. 38,39 The operators *X̂* and x~^j , along with their corresponding momenta satisfy canonical commutation relations as it was shown for the two-particle case in ref.^38^ and the *N* particle case in ref.^39^

In terms of new coordinates, the Hamiltonian is a sum of two independent parts, $Ĥ={Ĥ}_{\mathrm{col}}+{Ĥ}_{\mathrm{rel}}$ , where^21,40^

2 Ĥcol=−∂X22m+mΩv22X̂2−ω∂q22+ω2(q̂−gNX̂)2,Ĥrel=∑j=2N[−∂x̃j22mN+NmΩv22x~^j2]−∑j,k=2N∂x̃j∂x̃k2mN−mΩv22[∑j=2Nx~^j]2

The Hamiltonian ${Ĥ}_{\mathrm{col}}$ describes the collective vibration coupled to the cavity field, while ${Ĥ}_{\mathrm{rel}}$ defines the dynamics of the relative bond lengths, decoupled from the cavity and *X̂*. For what follows, the relations between the original operators *x̂*_*i*_’s and the new ones, *X̂* and x~^j ’s, will prove important,

3 x̂1=1N(X̂+∑j=2Nx~^j),x̂i=1N(X̂+∑j=2Nx~^j)−Nx~^i;i=2,...,N

**Analytical Solution of the Hybrid System.** After expanding the quadratic term ${\left(\mathrm{q̂}-g\sqrt{N}\mathrm{X̂}\right)}^{2}$ in eq 3 and introducing the dressed vibrational frequency Ω̅_v_^2^ = Ω_v_^2^ + ω_*d*_^2^ where $\omega_{d}=\sqrt{\mu_{0}^{2}N/\left(m\epsilon_{0}V\right)}$ is the so-called diamagnetic frequency or depolarization shift,^41,42^ ${Ĥ}_{\mathrm{col}}$ takes the form ${Ĥ}_{\mathrm{col}}$ = −∂_*X*_^2^/(2*m*) + *m*Ω̅_v_^2^ *X̂* ^2^/2 – ω∂_*q*_^2^/2 – ω*g* $\sqrt{N}\mathrm{q̂}\mathrm{X̂}$ . Then, ${Ĥ}_{\mathrm{col}}$ can be brought into canonical form through the transformation,

4 $$\begin{array}{c} \mathrm{X̂}=\frac{1}{\sqrt{m}}\left(-\frac{\Lambda {\mathrm{Q̂}}_{+}}{\sqrt{1+\Lambda^{2}}}+\frac{{\mathrm{Q̂}}_{-}}{\sqrt{1+\Lambda^{2}}}\right),\\ \mathrm{q̂}=-\sqrt{\omega }\left(\frac{{\mathrm{Q̂}}_{+}}{\sqrt{1+\Lambda^{2}}}+\frac{\Lambda {\mathrm{Q̂}}_{-}}{\sqrt{1+\Lambda^{2}}}\right) \end{array}$$

Here, the parameter $\Lambda =\alpha -\sqrt{1+\alpha^{2}}$ , with α = (ω^2^ – Ω̅_v_^2^)/(2ω_*d*_ω), quantifies the degree of mixing between the cavity and the collective molecular degrees of freedom.

After the transformation, ${Ĥ}_{\mathrm{col}}$ takes the canonical form ${Ĥ}_{\mathrm{col}}=\underset{l=\pm }{\sum }\left(-\partial_{Q_{l}}^{2}/2+\Omega_{l}^{2}{\mathrm{Q̂}}_{l}^{2}/2\right)$ . The normal-mode frequencies (polariton frequencies) of ${Ĥ}_{\mathrm{col}}$ are

5 $$\Omega_{\pm }^{2}=\frac{{\mathrm{\Omega ̅}}_{v}^{2}+\omega^{2}}{2}\pm \frac{1}{2}\sqrt{4\omega_{d}^{2}\omega^{2}+({\mathrm{\Omega ̅}}_{v}^{2}-\omega^{2}{)}^{2}}$$

The modes Ω_+_ and Ω_–_ are the upper and lower polaritons, respectively. The difference between the polariton frequencies Δ = Ω_+_ – Ω_–_ is the polariton gap. At resonance, ω = Ω_v_, the polariton gap is equal to the vacuum Rabi splitting (VRS) $\Omega_{R}=$ $\Delta_{\omega =\Omega_{v}}=$ ${\left(\Omega_{+}-\Omega_{-}\right)}_{\omega =\Omega_{v}}=$ $\sqrt{\omega_{d}^{2}\left(4\omega^{2}+\omega_{d}^{2}\right)}$ , the fundamental spectroscopic observable in hybrid light-matter systems. The normalized VRS, Ω_*R*_/Ω_v_ defines the regime of light-matter interaction. Typically, Ω_*R*_/Ω_v_ < 0.1 is defined as the strong coupling regime, while 0.1 ≤ Ω_*R*_/Ω_v_ < 1 is the ultrastrong coupling regime, where interaction terms beyond the rotating-wave approximation become important.^30,31^

From the canonical form of ${Ĥ}_{\mathrm{col}}$ follows the standard time-dependent solution for the polariton operators,

6 $${\mathrm{Q̂}}_{\pm }\left(t\right)={\mathrm{Q̂}}_{\pm }\left(0\right)\cos\left(\Omega_{\pm }t\right)+\frac{{\mathrm{Q̇̂}}_{\pm }\left(0\right)}{\Omega_{\pm }}\sin\left(\Omega_{\pm }t\right)$$

which allows us to obtain *X̂*(*t*) and *q̂*(*t*) via eq 4.

Next, we focus on the relative bond lengths x~^j ’s described by ${Ĥ}_{\mathrm{rel}}$ . A crucial observation is that the cavity mode is absent in ${Ĥ}_{\mathrm{rel}}$ , indicating that the evolution of x~^j ’s remains unaffected by the cavity. Consequently, we can obtain the general time-dependent solutions for x~^j(t) simply by using the uncoupled Hamiltonian ${Ĥ}_{0}=\overset{N}{\underset{i=1}{\sum }}\left[-\partial_{x_{i}}^{2}/\left(2m\right)+m\Omega_{v}^{2}{\mathrm{x̂}}_{i}^{2}/2\right]$ .

The standard time-dependent solution for the position operators given ${Ĥ}_{0}$ is *x̂*_*i*_(*t*) = *x̂*_*i*_(0) cos­(Ω_v_ *t*) + *p̂*_*i*_(0) sin­(Ω_v_ *t*)/(*mΩ*_v_). Substitution into the definition, x~^j=N−1/2(x̂1−x̂j) , yields

7 x~^j(t)=x̂1(0)−x̂j(0)Ncos(Ωvt)+p̂1(0)−p̂j(0)NmΩvsin(Ωvt)

where *j* = 2, ..., *N*. This shows that all relative bond lengths x~^j oscillate with the bare vibrational frequency Ω_v_. Thus, we have found the full eigenspectrum of Ĥ describing the *N* molecules coupled to the cavity, Ω_–_, {Ω_v_}_*N*−1_, Ω_+_. The spectrum has two polariton modes Ω_±_ and *N* – 1 modes Ω_v_ unaffected by the cavity, also known as dark states. We note that the eigenmodes and dynamics of the hybrid system could also be obtained by diagonalizing a Hessian matrix, as it was done in ref.^43^ in the rotating-wave approximation. Our solution reproduces the polariton modes obtained in^43^ under the rotating-wave approximation.

**Dynamics of the First Moments.** According to the Ehrenfest theorem, the first moments (e.g., ⟨*X̂*(*t*)⟩, ⟨*q̂*(*t*)⟩, ⟨*x̂*_*i*_(*t*)⟩, etc.) exactly equal the corresponding classical solutions in a harmonic system.^28,29^ Position-momentum uncertainty manifests itself only in higher-order observables. Thus, we employ classical mechanics to provide a clear description of the physical phenomena in our system at the level of the first moments under different initial conditions. With this in mind, we omit the operator hats for convenience from this point on.

To find *X*(*t*) and *q*(*t*), we substitute *Q*_±_(*t*) = *A*_±_ sin­(Ω_±_ *t* + ϕ_±_) [eq 6] into eq 4,

8 $$\begin{array}{c} X\left(t\right)=-\frac{\Lambda A_{+}\sin\left(\Omega_{+}t+\phi_{+}\right)}{\sqrt{m\left(1+\Lambda^{2}\right)}}+\frac{A_{-}\sin\left(\Omega_{-}t+\phi_{-}\right)}{\sqrt{m\left(1+\Lambda^{2}\right)}}\\ \frac{q\left(t\right)}{-\sqrt{\omega }}=\frac{A_{+}\sin\left(\Omega_{+}t+\phi_{+}\right)}{\sqrt{1+\Lambda^{2}}}+\frac{\Lambda A_{-}\sin\left(\Omega_{-}t+\phi_{-}\right)}{\sqrt{1+\Lambda^{2}}} \end{array}$$

where the amplitudes *A*_±_ and phases ϕ_±_ are determined by the initial conditions of the cavity-molecules system.

Next, from eq 7 we find the relative bond lengths, *x̃*_*j*_(*t*) = *A*_*j*_ sin (Ω_v_ *t* + ϕ_*j*_) (*j* = 2, ..., *N*), where *A*_*j*_ and ϕ_*j*_ are determined by the initial conditions. By substituting *X*(*t*) and *x̃*_*j*_(*t*) into eq 3 we can obtain the time-dependent bond lengths of each molecule, *x*_*i*_(*t*).

Having found the complete solution for the molecular ensemble coupled to the cavity, we will investigate the dynamics of collective and local molecular vibrations under different initial nonequilibrium conditions. Such initial conditions are motivated by an attempt to provide insights into cavity-modified chemical reactivity repeatedly reported in the literature.^3–8^ Out-of-equilibrium states naturally emerge within activated chemical complexes.^18^ The activated complex comprises vibrationally active molecules (reactants) that approach each other and realize a transient intermediate state, where one or more molecular bonds are stretched, compressed, or distorted.^18^ The displaced bonds can be thought of as harmonic oscillators displaced from equilibrium. The vibrational modes of the activated complex often dominate the reaction dynamics, making them critical degrees of freedom for reaction kinetics. The notion of an activated complex is crucial for chemical reactivity and plays an important role in the transition-state theory.^18^ However, we would like to clarify that with our model we do not try to match the initial conditions in transition-state theory, which operates in pure thermal equilibrium. In addition, transient atomic arrangements are important in roaming reactions.^44,45^ In what follows, we focus on the transient, out-of-equilibrium behavior of an ensemble of molecules under collective VSC in a cavity.

**Cavity-Induced Resonant Collective Beatings.** First, we look at the case of a fully excited ensemble, where all molecular bonds are initially stretched by *x*_0_ ≪ 1, i.e. *x*_*i*_(0) ≈ *x*_0_, while the initial velocities, *ẋ*_*i*_(0) = *v*_*i*_ satisfy the condition ∑_*i*_ *v*_*i*_ = 0, such that the velocities of individual molecules may not be zero, but the average vanishes. The cavity mode is considered to be unperturbed; i.e., *q*(0) = *q̇*(0) = 0. This is motivated by the polaritonic chemistry experiments with vacuum cavities, i.e., without external fields.^3–8^

Consequently, for the collective coordinate, we have $X(0)=\sqrt{N}x_{0}$ and *Ẋ*(0) = 0, and in combination with the initial conditions of the cavity, we find the phases ϕ_±_ = π/2 and the amplitudes $A_{-}=x_{0}\sqrt{mN/\left(\Lambda^{2}+1\right)}$ , *A*_+_ = – *ΛA*_–_ such that the collective coordinate in eq 8 reads

9 $$X\left(t\right)=x_{0}\sqrt{N}\left[\frac{\Lambda^{2}\cos\left(\Omega_{+}t\right)}{\Lambda^{2}+1}+\frac{\cos\left(\Omega_{-}t\right)}{\Lambda^{2}+1}\right]$$

In the strong coupling regime (not ultrastrong coupling^30,31^ ), the renormalization of the bare molecular vibrational frequency is negligible; i.e., Ω̅_v_ ≈ Ω_v_. Then, for the cavity resonantly coupled to the vibrations ω = Ω_v_, the mixing parameter is Λ = −1 and the two polariton modes contribute equally to the collective motion. Using the sum-to-product trigonometric identity, the collective vibration reads

10 $$X\left(t\right)=x_{0}\sqrt{N}\cos\left(\frac{\Omega_{+}+\Omega_{-}}{2}t\right)\cos\left(\frac{\Omega_{+}-\Omega_{-}}{2}t\right)$$

Under VSC, the collective coordinate oscillates with two frequencies: the high Σ = Ω_+_ + Ω_–_, and the low Δ = Ω_+_ – Ω_–_, which is equal to the polariton gap. Due to the polariton gap, the collective vibration exhibits slow beatings with a period *T* = 4π/Δ. At resonance, the beatings are inversely proportional to the VRS, *T*|_ω=Ω_v__ = 4π/Ω_*R*_. The VRS depends on the number of molecules collectively coupled as $\Omega_{R}∼\sqrt{N}$ . Thus, the VSC introduces an emerging time scale in the molecular dynamics, which depends on the collective properties of the coupled system. We note that cavity-induced collective beatings were first obtained with fully quantum mechanical simulations in ref.^21^ It is important to note that the influence of the collective beatings on the local molecular dynamics was not studied in.^21^ The local vibrational dynamics are the main focus of this work as we will see in what comes later.

In Figure 1, we show the evolution of the collective molecular coordinate. In Figure 1(a,b), we focus on resonant coupling, ω = Ω_v_. For strong coupling in Figure 1(a) (Ω_*R*_/Ω_v_ = 0.05, black line), we observe the emergence of slow beatings which significantly suppress collective vibrations at late times, while the short-time dynamics modifications are insignificant. The observed beating arises from the interference between two polariton frequencies, Ω_±_, which emerge when molecular vibrations are resonantly coupled to the cavity mode. This interference produces a fast and a slow oscillating modes, with the slowly oscillating mode defining the beating frequency.

**Figure 1:**
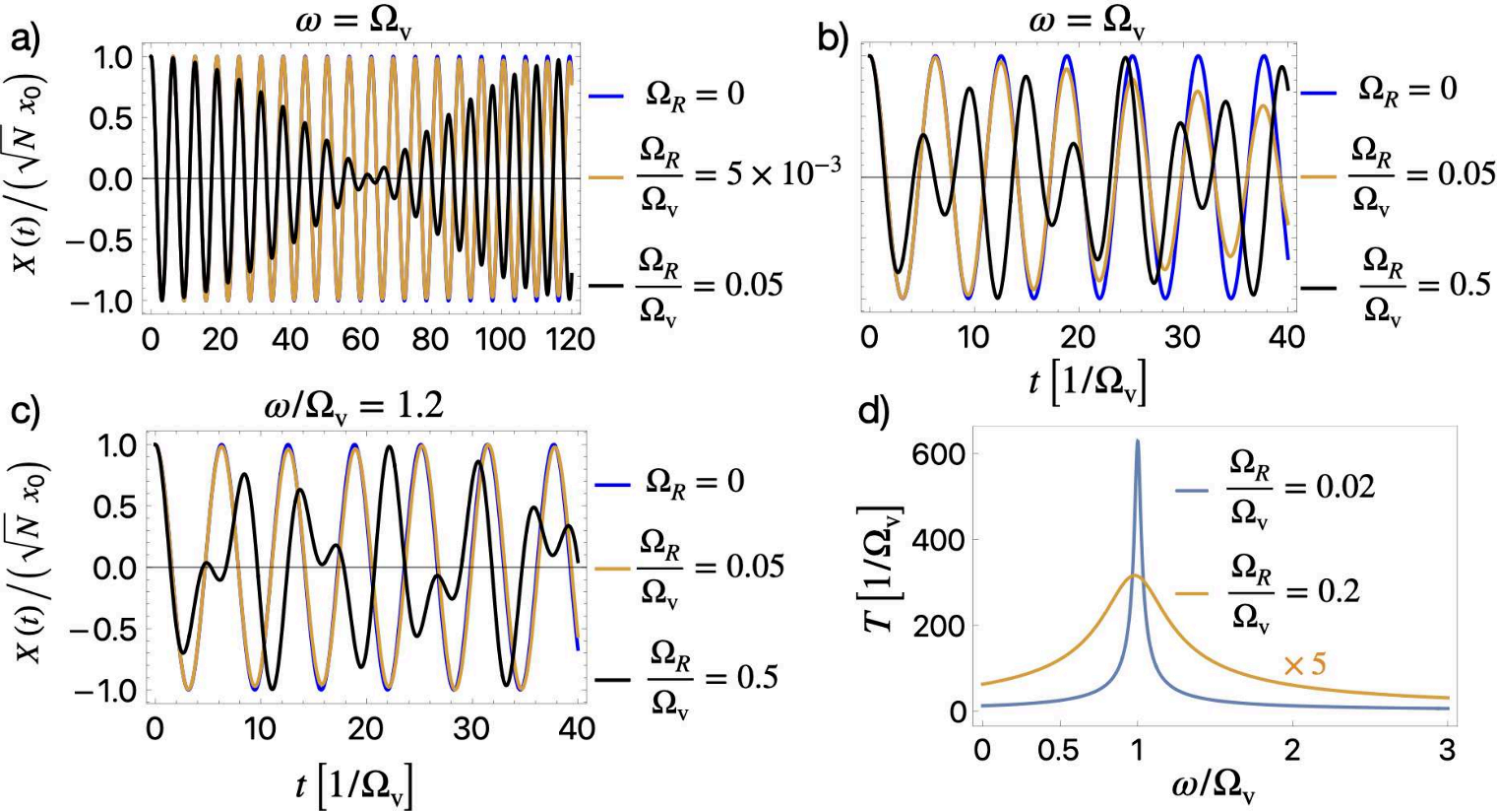
Evolution of the collective molecular coordinate, *X*(*t*) [see eqs 9 and 10] for different values of the coupling constant. (a) For resonant strong coupling (ω = Ω_v_, Ω_*R*_/Ω_v_ = 0.05, black), slow beatings emerge. For weaker coupling (Ω_*R*_/Ω_v_ = 0.005), the beating effect is negligible, and becomes apparent only on extremely long time scales. (b) For ultrastrong coupling (Ω_*R*_/Ω_v_ = 0.5, black) the cavity affects strongly also the short-time dynamics, as the collective vibration is suppressed during the first period of oscillation. For strong coupling, there is no visible effect on the short time scale. (c) Out of resonance (ω = 1.2Ω_v_), the collective vibrations are modified for ultrastrong coupling (Ω_*R*_/Ω_v_ = 0.5, black), while for strong coupling (Ω_*R*_/Ω_v_ = 0.05, orange) they remain unaffected. (d) Dependence of the beating period *T* for strong (Ω_*R*_/Ω_v_ = 0.02, blue) and ultrastrong (Ω_*R*_/Ω_v_ = 0.2, orange) couplings. For strong coupling, *T* peaks sharply around the resonance point, while for ultrastrong coupling, the beating period broadens and becomes asymmetric with respect to the resonance point.

In the same panel, for weaker coupling (Ω_*R*_/Ω_v_ = 0.005), no visible modification is observed, as the beatings become apparent only after extremely long times (not shown in the figure). For ultrastrong coupling (Ω_*R*_/Ω_v_ = 0.5) considered in Figure 1(b), even the short-time dynamics are modified by the cavity. This is a distinctive feature of ultrastrong coupling compared to strong. Next, in Figure 1(c), we detune the cavity from the vibrational mode by setting ω/Ω_v_ = 1.2 and observe that for strong coupling (Ω_*R*_/Ω_v_ = 0.05, orange line) no modification is observed. This striking finding highlights the importance of resonant coupling in modifying molecular vibrations in the strong coupling regime. This behavior aligns with the experimental observations in polaritonic chemistry, where the light-matter interaction is in the strong coupling regime, and modifications of the chemical reactivity occur mainly on resonance.^3–8^

Under ultrastrong coupling (Ω_*R*_/Ω_v_ = 0.5, black line), on the other hand, the collective vibrations are modified even off-resonance. Thus, we find that ultrastrong coupling has two important advantages compared to strong coupling: it allows for off-resonant and short-time modifications of the vibrational dynamics. These important features are also visualized in Figure 1(d) where the beating period *T* sharply peaks around the light-matter resonance for strong coupling (blue line), while for ultrastrong coupling, the curve broadens significantly. Thus, under ultrastrong coupling, molecular vibrations can be modified off-resonance as the beating period broadens around the cavity-molecule resonance (see Figure 1). This phenomenon could inspire new experimental approaches and deepen our understanding of vibrational dynamics in cavities.

Before continuing, we should discuss the values of the relevant experimental parameters required to observe the phenomena shown in Figure 1 in the strong and ultrastrong coupling regimes. Assuming a terahertz Fabry-Pérot cavity with frequency ω = 2π × 1 THz and effective cross-sectional area $A=1\;\mu m^{2}$ . The molecular mass is assumed to be *m* = 4 × 10^3^ *m*_*e*_, where *m*_*e*_ is the electron mass, the magnitude of the molecular dipole moment for simplicity is assumed to be one in units of fundamental charge μ_0_ = 1*e*, and the molecular vibration frequency is equal to the cavity mode Ω_v_ = ω = 2π × 1 THz. Given these parameters to reach collective strong coupling Ω_*R*_/Ω_v_ = 0.02, the number of collectively coupled molecules needs to be *N* ≈ 10^7^, while for ultrastrong coupling Ω_*R*_/Ω_v_ = 0.2 we need *N* ≈ 10^9^. Thus, the cavity-induced collective beatings we uncover, occur for the relevant parameters typically reported in polaritonic chemistry experiments.^9–11^ The same range of parameters will be used in what follows, where we focus on local vibrational dynamics.

**Resonant Suppression of Local Molecular Vibrations.** A key challenge in polaritonic chemistry is to understand whether cavity-induced modifications of collective molecular dynamics indeed affect the chemistry at the level of individual molecules. To address this, we investigate the evolution of the relative bond lengths {*x̃*_*j*_(*t*)}_*j*=2,···,*N*_ in order to access the individual local vibrations under the same initial conditions as previously, i.e., *x*_*i*_(0) ≈ *x*_0_ for *i* = 1, ..., *N*, ∑_*i*_ *v*_*i*_ = 0, and *q*(0) = *q̇*(0) = 0. In this case, the initial relative bond lengths and velocities are all zero *x̃*_*j*_(0) = 0, and thus the phases are all zero ϕ_*j*_ = 0 for *j* > 1. The initial velocities of the relative coordinates are *ṽ*_*j*_(0) = *N* ^–1/2^(*v*_1_ – *v*_*j*_) . Without loss of generality, we assume *v*_1_ = 0 and we have ${ṽ}_{j}(0)=-v_{j}/\sqrt{N}$ with *j* > 1. Thus, we find *x̃*_*j*_(*t*) = −*v*_*j*_ Ω_v_ $\sin(\Omega_{v}t)/\sqrt{N}$ for *j* > 1.

Since the sum over the initial velocities is zero, using eqs 3 and 9, we find the solutions for all molecules (*i* = 1, ..., *N*),

11 $$x_{i}\left(t\right)=\underset{\mathrm{polaritonic}}{\underset{︸}{x_{0}\left[\frac{\Lambda^{2}\cos\left(\Omega_{+}t\right)}{\Lambda^{2}+1}+\frac{\cos\left(\Omega_{-}t\right)}{\Lambda^{2}+1}\right]\;}}+\underset{\mathrm{bare},\mathrm{molecular}}{\underset{︸}{\frac{v_{i}}{\Omega_{v}}\sin\left(\Omega_{v}t\right)}}$$

Thus, vibrations of individual molecules are superpositions of vibration with bare molecular frequency Ω_v_ and collective molecular vibration, defined by polariton frequencies, Ω_±_. Importantly, the polaritonic contribution has no prefactor 1/*N*, which would make it negligible at the limit of *N* → ∞. This is one of the key findings of this work, showing that for nonequilibrium initial conditions, the polaritons modify dramatically the local molecular vibrations. Note that Λ depends on the particle density N/V only via $\omega_{d}=\sqrt{\mu_{0}^{2}N/\left(m\epsilon_{0}V\right)}$ which attains a finite value in the large *N* (thermodynamic) limit. The only situation in which the molecules are not affected by the polaritonic modes is if their initial vibrational velocities are so large that *v*_*i*_ ≫ *x*_0_Ω_v_.

In Figure 2, we visualize the local molecular dynamics under resonant VSC for different values of the VRS and different initial velocities. Figure 2(a) shows the emergence of beatings in local vibrations under strong coupling (Ω_*R*_/Ω_v_ = 0.07) that suppresses molecular vibrations on the long time scale. For ultrastrong coupling (Ω_*R*_/Ω_v_ = 0.5) in Figure 2(b), we observe that the short-time dynamics are also significantly affected by the collective oscillations. Next, in Figure 2(c-e) we provide the phase space visualization of the local vibrations for multiple molecules with different initial velocities and normalized VRS Ω_*R*_/Ω_v_ = 0.07. At short times [*t* = 5/Ω_v_, Figure 2(c)] all molecules seem to follow a circular trajectory around the origin. However, with time [*t* = 25/Ω_v_, Figure 2(d)], the trajectories gradually spiral toward the origin. This indicates that the vibrational energy is being transferred to the cavity. The cavity-induced resonant beatings force the molecular trajectories to spend more time closer to the phase-space origin, where their energy is low. This means that local molecular vibrations are effectively temporarily frozen [see Figure 2(e)]. For comparison, an uncoupled molecule [gray trajectories in Figure 2(c-e)] follows a fixed circular trajectory with constant total energy. Thus, from Figure 2, it becomes clear that the modifications of the collective vibrations *X*(*t*) are imprinted on the local molecular dynamics and drastically modify the individual molecular vibrations. This suggests a unique and general mechanism for modifying local molecular vibrations under resonant collective VSC in a cavity, particularly in the limit of many molecules. Thus, we expect that the cavity-induced resonant beating, can herald significant modifications in the out-of-equilibrium vibrational dynamics of molecules in activated complexes formed during chemical reactions.

**Figure 2:**
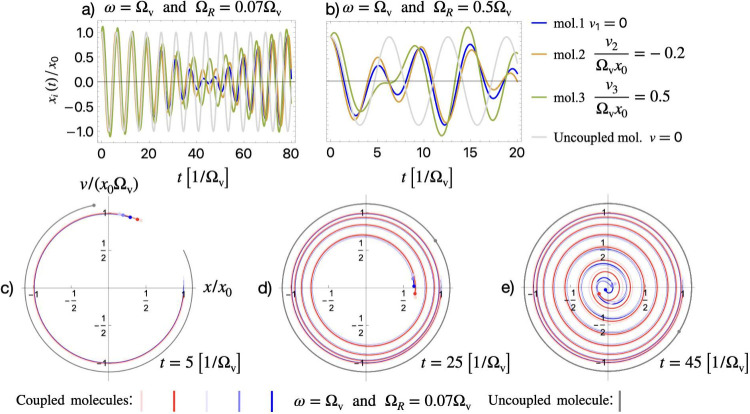
(a,b) Evolution of the vibrations of three representative molecules (out of the total of *N* molecules in the ensemble) with different initial velocities under resonant ω = Ω_v_ and collective VSC characterized by the normalized VRS Ω_*R*_/Ω_v_. (a) For strong coupling Ω_*R*_/Ω_v_ = 0.07, all molecules experience suppression of their local vibrations due to the cavity-induced resonant beatings at long time scales. (b) For ultrastrong coupling Ω_*R*_/Ω_v_ = 0.5, molecular vibrations are strongly modified at short times, even during the first oscillation. (c-e) Visualization of the phase space trajectories of local molecular vibrations for resonant coupling ω = Ω_v_ and normalized VRS Ω_*R*_/Ω_v_ = 0.07. (i) At short times, all molecules seem to follow a circular trajectory around the origin. (ii) At later times, however, the trajectories of coupled molecules spiral toward the phase space origin as their vibrational energy is transferred to the cavity. (iii) The cavity-induced resonant beatings force the molecules to spend more time close to the phase-space origin, where their vibrational energy is low. This means that the local molecular vibrations are effectively frozen during this time. For comparison, an uncoupled molecule (gray trajectory) follows a fixed circular trajectory with constant total energy.

**Modification of Local Vibrations in a Partially Activated Ensemble.** So far, we have considered the impact of cavity coupling on the vibrational dynamics of a fully excited molecular ensemble. However, in a more realistic scenario, only a fraction of the molecules are usually excited. Thus, we assume that there are *N*_ex_ excited molecules or, equivalently, a fraction β = *N*_ex_/*N* of the ensemble is displaced from equilibrium. For simplicity, we assume the excited molecules to be approximately stretched by the same small displacement *x*_*i*_(0) ≈ *x*_0_, ∀ *i* ∈ {1, ..., *N*_ex_} while *x*_*i*′_(0) = 0 for *i*′ ≠ *i*. In addition, we assume that all initial velocities are zero *ẋ*_*l*_(0) = *v*_*l*_ = 0 ∀*l* = 1,···,*N*. As previously, the cavity is not initially excited *q*(0) = *q̇*(0) = 0.

Correspondingly, the initial conditions for the collective vibration are $X(0)=\beta \sqrt{N}x_{0}$ and *Ẋ*(0) = 0. Combining them with the cavity initial conditions, we find the phases ϕ_±_ = π/2 and the amplitudes $A_{-}=\beta x_{0}\sqrt{mN/\left(\Lambda^{2}+1\right)}$ , *A*_+_ = – *ΛA*_–_ which determine the dynamical evolutions in eq 8. For the collective vibrations, we find

12 $$X\left(t\right)=\beta x_{0}\sqrt{N}\left[\frac{\Lambda^{2}\cos\left(\Omega_{+}t\right)}{\Lambda^{2}+1}+\frac{\cos\left(\Omega_{-}t\right)}{\Lambda^{2}+1}\right]$$

For finite β, the collective vibrations exhibit beatings shown in Figure 1(a) with β merely controlling the vibration amplitude. For zero initial velocities, the solutions for the relative bond length are *x̃*_*j*_(*t*) = *N* ^–1/2^[*x*_0_ – *x*_*j*_(0)] cos­(Ω_v_ *t*). Substituting the solutions for the collective vibration *X*(*t*) and the relative bond lengths *x̃*_*j*_(*t*) into eq 3, we obtain the solutions for the excited molecules,

13 $$\frac{x_{i}\left(t\right)}{x_{0}}=\frac{\beta \Lambda^{2}\cos\left(\Omega_{+}t\right)}{\Lambda^{2}+1}+\frac{\beta \cos\left(\Omega_{-}t\right)}{\Lambda^{2}+1}+\left(1-\beta \right)\cos\left(\Omega_{v}t\right)$$

where *i*∈{1, ..., *N*_ex_}. For the ground-state molecules in the ensemble

14 $$\frac{x_{i\prime }\left(t\right)}{x_{0}}=\frac{\beta \Lambda^{2}\cos\left(\Omega_{+}t\right)}{\Lambda^{2}+1}+\frac{\beta \cos\left(\Omega_{-}t\right)}{\Lambda^{2}+1}-\beta \cos\left(\Omega_{v}t\right)$$

where *i*′ ≠ *i*. From the above equations, we see that the local vibrations of both excited and ground-state molecules are affected by the polaritonic modes Ω_±_ that emerge due to the collective vibration *X*(*t*) [see eq 12]. At the same time, the local vibrations depend on the bare molecular frequency Ω_v_ as expected.

In Figure 3, we show the time evolution for the local vibrations of excited and ground-state molecules resonantly coupled to the cavity ω = Ω_v_, for different values of the activation ratio β = 0.05, 0.2 and different values of the collective VRS. For strong cavity-molecule coupling Ω_*R*_ = 0.07Ω_v_ in Figure 3(a), we observe that excited (initially stretched) molecules experience cavity-induced slow beatings, which substantially suppress their vibrations (black and cyan). Conversely, the initially not stretched molecules (blue and red) begin vibrating with time and exhibit beatings due to energy exchange with originally excited molecules. Thus, the cavity mediates this energy transfer, which is optimal under resonant coupling. This phenomenon appears for both 5% activation in the ensemble (β = 0.05) and for 20% (β = 0.2), which is more pronounced for the higher activation ratio β = 0.2, as intuitively expected. It is important to note that when the ensemble is partially vibrationally excited, the cavity mediates intermolecular energy exchange. In contrast, this phenomenon does not occur in a fully excited ensemble. This highlights the importance of the molecular initial states for the cavity-mediated dynamical correlation effects between the molecules in the ensemble.

**Figure 3:**
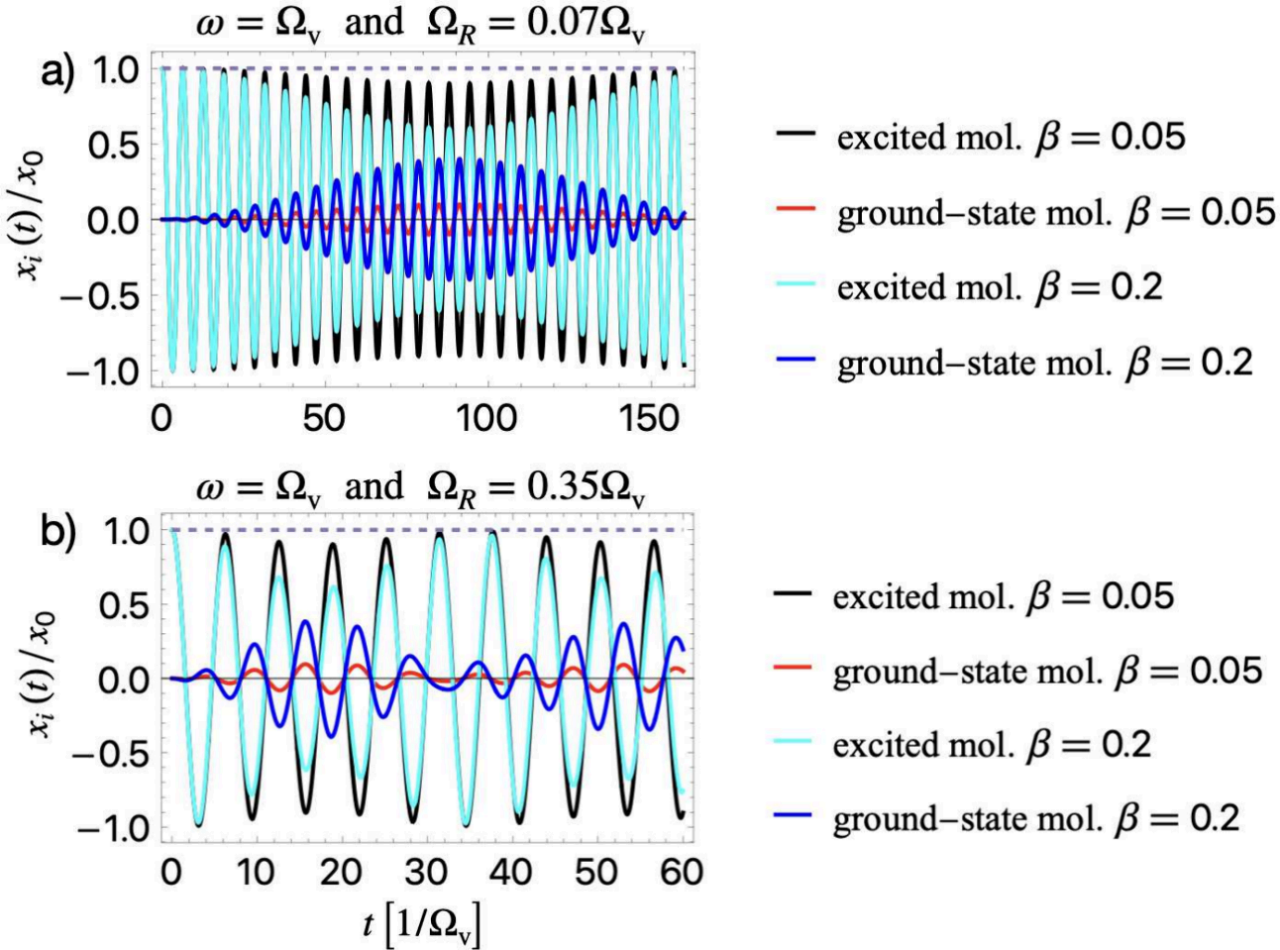
Evolution of the local molecular vibrations in a partially activated ensemble. (a) For resonant strong coupling, ω = Ω_v_, Ω_*R*_/Ω_v_ = 0.07, we observe that the excited molecules experience cavity-induced slow beatings, which suppress their vibrations (black and cyan). Conversely, the ground-state (red and blue) molecules begin vibrating with time and also exhibit beatings due to cavity-mediated energy exchange with originally excited molecules. (b) For ultrastrong coupling, Ω_*R*_/Ω_v_ = 0.35, the same dynamical behaviors as in (a) occur but with a faster rate, as the beatings in excited and ground-state molecules appear at much earlier times. The amplitude of the beating depends linearly on the activation ratio β = *N*_ex_/*N*.

In Figure 3(b), for ultrastrong coupling Ω_*R*_ = 0.35Ω_v_ the same phenomenon occurs but with a faster rate, as the beatings in the excited and ground-state molecules appear at much earlier times. Overall, Figure 3 demonstrates that the local vibrations of molecules in a partially activated ensemble are strongly affected under resonant VSC to a cavity. This is a significant result that broadens our understanding of the mechanisms that can affect local vibrations and reactivity in polaritonic chemistry. These changes are driven by strong cavity-molecule coupling, resulting in polariton formation, which highlights the intricate interplay between excited molecular ensembles and the cavity environment.

It is important to note that in the absence of excited molecules (*N*_ex_ = 0), only the solution in eq 14 applies, and for β = 0 becomes trivial *x*_*i*′_(*t*) = 0 meaning that we have no local vibrations as expected. In the opposite case of the fully activated ensemble (*N*_ex_ = *N*), only the solution in eq 13 is true, and for β = 1 we recover the result in eq 11 for the case of zero initial velocities *v*_*i*_ = 0. This demonstrates the consistency of our solution.

**Isotropic Ensemble.** So far in this work, we considered the simple case of a fully oriented ensemble where all the molecules are oriented along the cavity field polarization. In our one-dimensional model, an approximate representation of an isotropic ensemble which has zero total dipole, can be considered by having half of the molecular dipoles in the direction of the field polarization (oriented) and half of the dipoles in the opposite direction (antioriented). Thus, the Hamiltonian takes the form

15 $${Ĥ}_{\mathrm{iso}}=\overset{N}{\underset{i=1}{\sum }}\left[-\frac{1}{2m}\frac{\partial^{2}}{\partial x_{i}^{2}}+\frac{m\Omega_{v}^{2}}{2}{\mathrm{x̂}}_{i}^{2}-\frac{1}{2m}\frac{\partial^{2}}{\partial s_{i}^{2}}+\frac{m\Omega_{v}^{2}}{2}{ŝ}_{i}^{2}\right]-\frac{\omega \partial_{q}^{2}}{2}+\frac{\omega }{2}{\left(\mathrm{q̂}-g\overset{N}{\underset{i=1}{\sum }}{\mathrm{x̂}}_{i}+g\overset{N}{\underset{i=1}{\sum }}{ŝ}_{i}\right)}^{2}$$

The operators {*x̂*_*i*_, ∂_*x*_*i*__} describe the molecules oriented along the field, while the operators {*ŝ*_*i*_, ∂_*s*_*i*__} describe the antioriented ones. This can be understood from the opposite sign in their respective light-matter couplings. Note that all molecules have the same mass *m* and the same vibrational frequency Ω_v_. The cavity mode couples only to the collective coordinates of the aligned and the antialigned molecules respectively *X̂* = *N* ^–1/2^ ∑_*i*_ *x̂*_*i*_ and *Ŝ* = *N* ^–1/2^ ∑_*i*_ *ŝ*_*i*_, while the relative bond lengths x~^j=N−1/2(x̂1−x̂j) and s~^j=N−1/2(ŝ1−ŝj) with *j* = 2, ..., *N* decouple from the cavity mode. This can be seen straightforwardly in the new coordinate frame,

16 Ĥiso=−∂X22m+mΩv22X̂2−∂S22m+mΩv22Ŝ2−ω∂q22+ω2(q̂−gNX̂+gNŜ)2+Ĥrel(x~^j,s~^j)

In the above, the term Ĥrel(x~^j,s~^j) does not contain the cavity mode. Next, we introduce two new collective degrees of freedom that connect the oriented and the antioriented molecular subsystems, namely, a symmetric linear combination *r̂* = (*X̂* + *Ŝ*)/√2 and an antisymmetric one *R̂* = (*X̂* – *Ŝ*)/√2. The symmetric coordinate *r̂* decouples, and the collective coupling of the whole isotropic ensemble to the cavity is captured by the antisymmetric coordinate *R̂*. The coupling of *R̂* to the cavity has exactly the same form as the Hamiltonian ${Ĥ}_{\mathrm{col}}$ [see eq 2] in the fully oriented ensemble,

$${Ĥ}_{\mathrm{col}-iso}=-\frac{\partial_{R}^{2}}{2m}+\frac{m\Omega_{v}^{2}}{2}{\mathrm{R̂}}^{2}-\frac{\omega \partial_{q}^{2}}{2}+\frac{\omega }{2}{\left(\mathrm{q̂}-g\sqrt{2N}\mathrm{R̂}\right)}^{2}$$ 17

From the above result, it becomes evident that the cavity-induced dynamics that we uncovered for the case of the fully oriented ensemble, all transfer straightforwardly to the isotropic ensemble as well, for the antisymmetrically correlated coordinate between the antialigned subsystems. Thus, the cavity-induced resonant and collective effects do not vanish for an isotropic ensemble coupled to the cavity. The explicit dynamical behavior of the oriented and the antioriented subsystems under different initial conditions will be discussed in a future publication.

**Discussion and Outlook.** A fundamental mechanistic understanding of the observed VSC effects in polaritonic chemistry would be a significant leap forward, as it would provide a minimal-waste form of heterogeneous catalysis.^10^

We propose a unique and general mechanism for modifying local molecular vibrations under resonant collective VSC in a cavity, particularly in the limit of many molecules. We discover a cavity-induced collective and resonant beating phenomenon that emerges in the strong coupling regime, heralding significant modifications in the local vibrational dynamics of molecules; excited in the intermediate out-of-equilibrium complexes formed during chemical reactions.

In our model, we assume that during a chemical reaction, a small fraction of reactant molecules forms an activated complex in which a specific bond is initially displaced from its equilibrium position. Under these initial conditions the collective motion of the system exhibits beatings that periodically suppress the motion of individual bonds. Consequently, vibrational energy is suppressed, limiting the complexs ability to rearrange along other reaction pathways. If the beating period is comparable to the lifetime of the activated complex, the likelihood of it converting into products decreases, resulting in a measurable slowdown in the reaction rate. This effect spreads through the entire ensemble via cavity-mediated energy exchange, even when only a small fraction (1 – 5%) of molecules are initially excited.

These phenomena are driven by strong cavity-molecule coupling, resulting in polariton formation, which highlights the intricate interplay between excited molecular ensembles and the cavity environment. The beating period is inversely proportional to the VRS and thus depends on the number of collectively coupled molecules. The beating period exhibits a pronounced peak at the cavity-molecule resonance. This is the origin of the resonant suppression of local vibrations.

In the ultrastrong coupling regime, where the counter-rotating terms are important,^30–32^ the cavity modifies the local vibrations at short time scales and our theory makes an experimentally testable prediction: under ultrastrong coupling, molecular vibrations can be modified off-resonance as the beating period broadens around the cavity-molecule resonance (see Figure 1). This phenomenon could inspire new experimental approaches and deepen our understanding of vibrational dynamics in cavities. In addition, pulsed laser-assisted excitations in molecules can engineer activated complexes.^46,47^ This straightforward method offers a controlled way to test the proposed mechanism, potentially with cold and clean molecular systems.^48–50^

A key aspect of our work is that the resonant suppression of vibrations requires *only* a small fraction of excited molecules in the ensemble (∼1–5%). Importantly, the energy flows from the initially excited molecules into the ground-state molecules, which then start to oscillate. Thus, the cavity mediates an energy exchange and interactions between the excited and ground-state molecules.

A comprehensive understanding of the cavity-induced modification of chemical reactivity and dissociation dynamics requires the anharmonicity of the molecular potential to be included. We intend to investigate the dynamics of Morse oscillators^51^ collectively coupled to the cavity mode in an upcoming publication. We anticipate that the resonant cavity-mediated energy transfer between the molecules that we uncover here will substantially modify the dissociation dynamics in the collective regime. It is important to highlight that the anharmonic terms will couple the collective Hamiltonian ${Ĥ}_{\mathrm{col}}$ and intermolecular bond lengths ${Ĥ}_{\mathrm{rel}}$ , introducing an additional layer of complexity. Further, the inclusion of anharmonicities will introduce nonlinear dynamical phenomena which go beyond the present formalism. At the same time, for anharmonic systems, the dynamics of first moments cannot be fully captured with the semiclassical approach, because of the quantum revivals emerging on the long time scale, as mentioned in refs. 26,27 For anharmonic systems, only the early time dynamics can be approximately captured with the semiclassical approach.^29^

At this point, we would like to mention a relevant work which also studied VSC from an out-of-equilibrium perspective utilizing classical dynamics.^52^ This numerical study considered a model including vibrational anharmonicities and bending modes. It was found that the dissociation dynamics of *N* perfectly oriented classical molecules can be suppressed by strong coupling to a cavity.^52^ In connection with our results, the resonant cavity-induced collective beatings were not reported there.

The role of molecular orientation and disorder^53,54^ within our model needs to be investigated further. We expect that the cavity-induced beatings will emerge even in the presence of weak disorder because the light-matter interaction will dominate. At the same time, from the generalization of our model to isotropic ensembles we anticipate that for ensembles where half of the molecules are oriented parallel and half antiparallel to the cavity field polarization will also experience collective beatings and under different out-of-equilibrium initial conditions, VSC will modify the local molecular vibrations. In addition, we comment on the role of thermal disorder, which typically increases the random motion and energy distribution among molecules. In a more random state, we anticipate that the beatings will be suppressed, due to the suppression of the collective vibrations. However, a conclusive answer requires further investigation, as molecular orientations also depend on the thermal fluctuations in the ensemble.

This study advances the understanding of VSC and its implications in polaritonic chemistry. We underline the importance of out-of-equilibrium molecular states in hybrid cavity-molecule systems for local vibrational dynamics in activated complexes. Our findings suggest that the molecular out-of-equilibrium states seed cavity-induced modulation of chemical reactivity under VSC, potentially explaining chemical behavior in polaritonic systems. Further, we showcase that resonant dynamical phenomena can be captured with classical formalism, thus opening a path for understanding resonant collective phenomena in polaritonic chemistry with classical dynamics simulations. We thus provide a foundation for further exploration of cavity-modulated chemical reactions. The ability to experimentally verify these predictions opens avenues for developing tailored chemical systems that leverage VSC to control chemical reactions and properties.
